# Selection of Fermentation Supernatant from Probiotic Strains Exhibiting Intestinal Epithelial Barrier Protective Ability and Evaluation of Their Effects on Colitis Mouse and Weaned Piglet Models

**DOI:** 10.3390/nu16081138

**Published:** 2024-04-12

**Authors:** Solomon Abrehame, Man-Yun Hung, Yu-Yi Chen, Yu-Tse Liu, Yung-Tsung Chen, Fang-Chueh Liu, Yu-Chun Lin, Yen-Po Chen

**Affiliations:** 1 Department of Animal Science, National Chung Hsing University, 145 Xingda Road, South District, Taichung City 402, Taiwan; 2 The iEGG and Animal Biotechnology Center, National Chung Hsing University, 145 Xingda Road, South District, Taichung City 402, Taiwan; 3 Ethiopian Agricultural Authority, Ministry of Agriculture of Ethiopia (MoA), P.O. Box 62347, Addis Ababa 1000, Ethiopia; 4 Department of Food Science, National Taiwan Ocean University, 2 Beining Road, Zhongzheng District, Keelung City 202, Taiwan; 5 Animal Nutrition Division, Taiwan Livestock Research Institute, Ministry of Agriculture, 112 Farm Road, HsinHua District, Tainan City 712, Taiwan; 6 Fisheries Research Institute, Ministry of Agriculture, 199 Hou-Ih Road, Keelung City 202, Taiwan

**Keywords:** cell-free supernatant, intestinal barrier function, probiotic, postbiotic

## Abstract

The intestinal epithelial barrier can prevent the invasion of pathogenic microorganisms and food antigens to maintain a consistent intestinal homeostasis. However, an imbalance in this barrier can result in various diseases, such as inflammatory bowel disease, malnutrition, and metabolic disease. Thus, the aim of this study was to select probiotic strains with epithelial barrier-enhancing ability in cell-based model and further investigate them for their improving effects on colitis mouse and weaned piglet models. The results showed that selected specific cell-free fermentation supernatants (CFSs) from *Ligilactobacillus salivarius* P1, *Lactobacillus gasseri* P12, and *Limosilactobacillus reuteri* G7 promoted intestinal epithelial cell growth and proliferation, strengthening the intestinal barrier in an intestinal epithelial cell line Caco-2 model. Further, the administration of CFSs of *L. salivarius* P1, *L. gasseri* P12, and *L. reuteri* G7 were found to ameliorate DSS-induced colitis in mice. Additionally, spray-dried powders of CFS from the three strains were examined in a weaned piglet model, only CFS powder of *L. reuteri* G7 could ameliorate the feed/gain ratio and serum levels of D-lactate and endotoxin. In conclusion, a new potential probiotic strain, *L. reuteri* G7, was selected and showed ameliorating effects in both colitis mouse and weaned piglet models.

## 1. Introduction

The intestinal tract is a vital organ responsible for digestion and absorption, and it also acts as a barrier to prevent harmful substances such as pathogenic bacteria and toxins from entering the body. The intestinal epithelial barrier is composed of a single layer of epithelial cells that form a tight structure through the interaction of related surface proteins, creating a barrier that blocks harmful substances and regulates the transport of nutrients [1]. Dysfunction of the intestinal epithelial barrier has been linked to several diseases, including inflammatory bowel disease, metabolic disease, and autoimmune diseases [2,3,4]. As a result, the intestinal barrier has become a target for the treatment of these conditions [5,6].

Probiotics are live microorganisms that provide health benefits when consumed in a certain amount. They can inhibit or eliminate pathogenic bacteria in the gut, enhance the intestinal epithelial barrier, and regulate host immunity [7,8]. However, the use of probiotics is not without challenges. These include maintaining their activity, understanding their complex mechanism of action, and identifying potential risks such as bacterial infection, gene transfer of antibiotic resistance, and excessive immune response [9]. In addition, probiotics generate a significant volume of by-products during the fermentation process, posing a substantial challenge for waste management in manufacturing facilities.

Postbiotics, on the other hand, are soluble metabolites secreted by live bacteria or released after bacterial lysis, and their definition by the International Scientific Association of Probiotics and Prebiotics (ISAPP) is “preparation of inanimate microorganisms and/or their components that confers a health benefit on the host” [10,11]. They have been shown to provide health benefits by modifying host cell metabolic pathways [12]. In comparison to live bacteria, postbiotics have more versatile characteristics, including a clear chemical structure, safe dosage parameters, and a lack of the potential risks associated with live bacteria. As a result, postbiotics have emerged as a promising strategy for promoting healthy food and treating diseases [13,14,15]. The cell-free supernatant (CFS) produced by bacterial fermentation contains postbiotics secreted by the indicated probiotics, which have been shown to have protective effects on the intestine similar to those of live bacteria, suggesting that their active substances come from soluble molecules in the supernatant that can regulate the secretion of inflammatory factors or activate related immune cells [16]. By using postbiotics, it is possible to avoid the doubts and disposal burdens associated with live bacteria while still achieving desired health and therapeutic effects.

The goal of this study was to screen probiotic fermentation supernatants that protect the intestinal epithelial barrier through cell experiments and evaluate their therapeutic and health effects on animal models with impaired intestinal barriers, including dextran sodium sulfate (DSS)-induced colitis in mice, as well as a weaned piglet model. This will help to design a rational screening platform for selecting postbiotics/probiotics for further application to ameliorate intestinal barrier-related disorders.

## 2. Materials and Methods

### 2.1. Bacterial Strains and Fermentation Supernatant Preparation

A total of 34 candidate bacterial strains, which were kindly provided from strain bank of the Animal Nutrition Division, Taiwan Livestock Research Institute, Ministry of Agriculture, Taiwan, were numbered as P1–P26 and G1–G8. These strains were cultured in de Man Rogosa and Sharpe (MRS) broth (Becton Dickinson and Company Difco^TM^, Sparks, MD, USA) at 37 °C for 24 h. Pure colonies of each strain were cryopreserved after culture and used for the remainder of the study. The bacteria were identified using amplification and sequencing of the conserved regions of microbial 16S rDNA and comparison with the NCBI database (Genomics Co., Ltd., Taipei, Taiwan). CFSs were prepared by centrifuging the bacterial fermentation liquid (OD_600_: 1.0) at 1500× *g* for 10 min (Megafuse 16R centrifuge, ThermoFisher, Waltham, MA, USA) and collecting the supernatants. The supernatants were adjusted to pH7 by 0.1 M NaOH (Sigma, St. Louis, MO, USA) and further filtered with a 0.22 μm filter (non-Pyrogenic, Pall Corporation, Port Washington, NY, USA), followed by aliquoting them into microcentrifuge tubes for storage at −80 °C until use.

### 2.2. Cell Culture

Human intestinal epithelial cell line Caco-2 was obtained from the Bioresource Collection and Research Center, Food Industry Research and Development Institute (Hsinchu, Taiwan), and maintained in Dulbecco’s Modified Eagle’s Medium (DMEM, 4.5 g/L D-glucose and L-glutamine, Gibco, Thermo Fisher Scientific Inc., Paisley, Scotland, UK) with or without 10% fetal bovine serum (FBS, Gibco, Thermo Fisher Scientific Inc., Paisley, Scotland, UK), 1% sodium pyruvate (Gibco, Thermo Fisher Scientific Inc., Paisley, Scotland, UK), 1% holo-transferrin (human origin, Sigma, Steinheim, Germany), and antibiotic–antimycotic (Gibco, Thermo Fisher Scientific Inc., Paisley, Scotland, UK) with a regular cell culture protocol.

### 2.3. CFS Treatment on Intestinal Cell Viability and Proliferation In Vitro

The adequately maintained Caco-2 cells were inoculated onto a 24-well plate at a concentration of 1 × 10^5^ cells per well and incubated at 37 °C with 5% CO_2_ overnight. After incubation, the medium was replaced with fresh one with candidate bacterial CFS at indicated concentrations (0.2%, 1% and 5%) for 24 h, and the supernatant was collected and stored at −80 °C for subsequent analysis. The MRS medium from the same batch without fermentation by bacteria was used at the indicated concentration as a control. The effects of the CFS against DSS-damaged cells (DSS, MP Biomedicals, Montreal, QC, Canada) were evaluated by co-culturing the cells with indicated concentrations (0.2%, 1% and 5%) of probiotic CFS with or without 3% DSS for 24 h [17]. Cell viability was measured using an MTT assay according to Kumar et al. [18], and cell proliferation was measured using a BrdU assay referring to the method of Adnan et al. [19].

### 2.4. Caco-2 Intestinal Epithelial Monolayer Preparation and Transepithelial Electrical Resistance (TEER) Measurement

According to Chen et al. [17], the Caco-2 cells were inoculated at 10^5^ cells onto a 12-well permeable membrane (Transwell Permeable Supports, 12 mm insert, 12 well plate, 3.0 μm polycarbonate membrane, Corning, NY, USA). The culture medium in both the basolateral and apical sites of the membrane were replaced every 2–3 days during 21–28 days of incubation. The transepithelial electrical resistance (TEER) was measured by using an EVOM2 epithelial voltohmmeter (World Precision Instruments, Hertfordshire, UK) with an STX2 probe (World Precision Instruments) according to the manufacturer’s instructions. The Caco-2 monolayers were ready to use until the TEER values were greater than 300 Ωcm^2^. The Caco-2 monolayers were co-cultured with indicated bacterial CFS at the apical site with or without 3% DSS at both apical and basolateral sites for 24 h, according to Peng et al. [20] and Chen et al. [17]. After incubation, TEER values were measured based on the above description.

### 2.5. DSS-Induced Colitis Mice

The mouse experiment was approved by the Institutional Animal Care and Use Committee (IACUC) of National Chung Ching University, Taiwan (Approval number: 109-027), and handling of the mice followed the animal welfare regulations of Taiwan and the guidelines for the management and use of experimental animals issued by the Ministry of Agriculture, Taiwan. Eight-week-old female C57BL/6 mice (BioLASCO, Taipei, Taiwan) were adapted for a week before the experiment. They were given a sterilized standard diet (Laboratory Autoclavable Rodent Diet 5010, Lab Det, PMI Nutrition, St. Louis, MO, USA) and water ad libitum in an environment with temperature 23–25 °C, humidity 40–60%, 12 h day/night cycle. The mice were randomly divided into five groups (n = 10 in each group): (1) control, the mice were fed with sterilized MRS medium; (2) DSS control, the mice were fed with sterilized MRS medium and colitis was induced by DSS; (3) P1-CFS, the mice were fed with *L. salivarius* P1-CFS and colitis was induced by DSS; (4) P12-CFS, the mice were fed with *L. gasseri* P12-CFS and colitis was induced by DSS; and (5) G7-CFS, the mice were fed with *L. reuteri* G7-CFS and colitis was induced by DSS. The mice were fed with freshly prepared sterilized MRS medium or the indicated CFS by oral gavage at 200 μL/mouse daily for 14 days, and then Colitis was induced in the last 7 days of the feeding period by adding 2.5% DSS to the drinking water, with water replaced every 2 days with freshly prepared solution. The clinical symptoms were recorded during the experiment, and the mice were sacrificed and sampled at the end of the experiment. Mice sacrifice was done humanely by using isoflurane-soaked cotton for gas anesthesia. Once they were euthanized, blood samples were drawn by cardiac puncture, and colon tissue was collected after the dissection of the mice. A section approximately 1 cm long of colon tissue was cut out, rinsed twice with sterilized PBS, and soaked in 10% formaldehyde (10% in Aqueous Phosphate Buffer, Avant Performance Materials, Inc., Center Valley, PA, USA).

### 2.6. Disease Activity Index

According to the method developed by Sann et al. [21] and Wirtz et al. [22,23], the disease activity index (DAI) involved combining the scores for weight loss, diarrhea, and fecal occult blood to obtain a total score, which could reflect the overall severity of the disease. The mouse body weight loss score was graded as follows: 0 indicating the body weight loss was <2%, 1 indicating the body weight loss was ≥2% to <5%, 2 indicating the body weight loss was ≥5% to <10%, 3 indicating the body weight loss was ≥10% to <15%, and 4 indicating the body weight loss was ≥15%. Fecal samples were collected and analyzed with a commercially available kit for detecting occult blood in feces (Hemoccult Sensa FOBT (Fecal Occult Blood Test) Slides, Beckman Coulter, Brea, CA, USA). The presence of fecal occult blood was graded on a scale of 0 to 3, with 0 indicating the absence of occult blood, 1 indicating a positive result on the kit, 2 indicating the presence of blood visible to the naked eye in the feces, and 3 indicating the presence of rectal bleeding visible around the anus. Each grading interval represents an increment of 0.5 points. The feces were graded on a scale of 0 to 3, with 1 indicating normal consistency, 2 indicating soft and muddy feces, and 3 indicating severe diarrhea to the point where the feces could not be formed into a coherent shape. As with the occult blood grading, each interval on this scale represents an increment of 0.5 points.

### 2.7. Histological Evaluation of the Colon

The intestinal tissue sections were assessed using hematoxylin and eosin (H&E) staining, following the method established by Erben et al. [24]. The histological sections were evaluated by at least 2 veterinary histopathologists (Animal Technology Research Center, Agricultural Technology Research Institute, Miaoli County, Taiwan) in a blinded manner without knowing any experimental design, based on the methods of Dieleman et al. and Erben et al. [24,25], including four indicators: inflammation, ulceration, edema, and the overall extent of disease. Inflammation and ulceration were scored on a scale of 0 to 3, with 0 indicating the absence of inflammation or ulceration and 3 indicating the most severe inflammation or ulceration [24,25]. The extent of disease was quantified as a percentage of the area affected, with scores of 1, 2, 3, or 4 corresponding to disease affecting 1–25%, 26–50%, 51–75%, and 76–100% of the area, respectively. Edema was scored on a scale of 0 to 3, according to Vlantis et al. [26], with 0 indicating no edema and 3 indicating severe edema.

### 2.8. Determination of Serum Cytokines

The blood samples were placed in a microtainer tube (Becton Dickinson and Company, Difco™, Sparks, MD, USA) and centrifuged at 3200× *g* for 10 min at 4 °C to isolate the serum. The assay was performed using an enzyme-linked immunosorbent assay (ELISA) to measure levels of interleukin-6 (IL-6) and tumor necrosis factor-α (TNF-α) using commercial kits (DuoSet, R&D System Inc., Minneapolis, MN, USA) according to manufacturer’s instructions.

### 2.9. In Vivo Intestinal Permeability Assay

Barrier function of the intestine was assessed using in vivo permeability assays with fluorescein isothiocyanate-dextran (FITC-D), following the method of Baxter et al. [27]. Four hours before sacrifice, the mice were given FITC-D (Sigma) at a concentration of 60 mg/100 g of body weight. At the time of sacrifice, 100 μL of serum was collected and transferred to a 96-well black plate, and the fluorescence intensity of the samples was measured using a fluorometer (Infinite 200 PRO, Mannedorf, Switzerland) with excitation light at 492 nm and emission light at 525 nm [27].

### 2.10. Effects of CFS on Weaned Piglets

The pig experiment was approved by the IACUC of Taiwan Livestock Research Institute, Ministry of Agriculture, Taiwan (Approval number: 109-40). Forty-eight 4-week-old cross-bred (Landrace × Yorkshire) weaned piglets from the Animal Nutrition Division, Taiwan Livestock Research Institute, Council of Agriculture, Executive Yuan, Taiwan, were used. The weaned piglets were randomly divided into six groups (n = 8 in each group) according to their average weaning weight (6.5 kg) and sex. Each group had four pens, with two pigs (one male and one female) per pen. The experimental groups included a negative control group that received the basal diet (Control), a positive control group that received the basal diet plus 0.5 kg antibiotics per ton of feed (Colistin-120, China Chemical & Pharmaceutical Co., Ltd., Taipei, Taiwan) (Antibiotic), and four treatment groups that received the basal diet with indicated amounts of CFS powders from P1 (5%), P12 (5%), G7(L) (0.5%), and G7(H) (5%), respectively. The feed formulas for all groups were in accordance with the NRC basal diet standard. All piglets were raised in the same controlled environment and were given the experimental feed for 6 weeks. Throughout the experiment, the piglets had access to clean drinking water at all times, and their body weight and feed intake were measured weekly. Blood samples were collected from the jugular vein on the second day at the beginning and end of the experiment for analysis.

### 2.11. Preparation of CFS Powder for Feedstuffs

The cryopreserved strains were thawed and cultured in MRS medium at 37 °C for 24 h for two rounds and then inoculated into MRS medium at a concentration of 1% and incubated at 37 °C for 24 h in laboratory scale. The cultured bacterial suspensions were then inoculated into a 30 L fermentation tank (Biotop, Taipei, Taiwan) for further fermentation, during which the temperature, pH value, and defoaming operations were controlled under fixed conditions. The fermentation was completed by centrifuging the fermentation suspension to remove the bacterial pellet, resulting in about 20 L of CFS. The obtained CFSs were concentrated using a Hei-VAP decompression concentrator (Heidolph Instruments, Schwabach, Germany), achieving a 6- to 8-fold concentration. The concentrated CFS was mixed with corn starch as an excipient and freeze dried to produce a dry powder that was used to formulate the diets of each experimental group.

### 2.12. Piglet Body Weight and Growth Performance

During the experiment, the weaning piglets were weighed and recorded on a weekly basis. The daily feed intake of each pen was calculated to determine the feed-to-weight gain ratio (F:G) and the feed conversion rate (FCR) using the following equations [28]:

Feed efficiency = total weight gain (kg)/total feed intake (kg)

Feed conversion rate = total feed intake (kg)/total weight gain (kg)

### 2.13. Measurement of Cytokine Production and Endotoxin Levels in Serum

The collected blood samples were centrifuged at 1500× *g* for 15 min at 4 °C in order to obtain serum. The serum was then used to test for the cytokine levels using the MILLIPLEX^®^ multiplex detection platform (Millipore, Merck, KGaA, Darmstadt, Germany). The serum endotoxin levels were examined using a commercial kit (Pierce^TM^ Chromogenic Endotoxin Quant Kit, Thermo Scientific) according to manufacturer’s instruction.

### 2.14. Statistical Analysis

The statistical analysis was conducted by using SAS 9.4 (Statistical Analysis System software 9.4, SAS Institute Inc., Cary, NC, USA). The main method used in the analysis was the least squares means of the general linear model (GLM), followed by Ducan’s multiple range significance test. These statistical methods were used to compare the mean values of each group and identify significant differences; hence, a significance level of *p* < 0.05 was used.

## 3. Results

### 3.1. Preliminary Screening of Potential CFSs That Could Enhance Intestinal Epithelial Cell Viability

First, 34 candidate bacterial strains from the bacteria bank of the Taiwan Livestock Research Institute, Taiwan, were used for evaluating the effect of their CFSs on intestinal epithelial cell viability with or without serum in cell culture condition. The CFSs did not show any visible viability differences in cells in a complete medium at a concentration of 1% (Figure S1a). In a serum-free medium, the effect of the CFSs on Caco-2 cell viability was more apparent, with half of the supernatants showing a significant increase in cell viability (Figure S1b). From this, 18 of the most effective CFSs were chosen for a second experiment for confirmation, where the selected supernatants were co-cultured with Caco-2 cells at concentrations of 0.2%, 1%, and 5% in media with or without serum. Based on the overall consideration, strains P1, P12, P21, G5, and G7 were selected for further evaluation in cell culture systems (Figure S2). The selected strains were identified based on their full sequence of 16S rDNA, and the results of sequence comparison revealed that P1 and G5 belonged to *Ligilactobacillus salivarius*, P12 and P21 belonged to *Lactobacillus gasseri*, and G7 belonged to *Limosilactobacillus reuteri*. These CFSs would be referred to as P1, P12, P21, G5, and G7 in the remainder of this study.

### 3.2. Effect of Selected CFSs on Intestinal Epithelial Cell Proliferation

**Figure 1:**
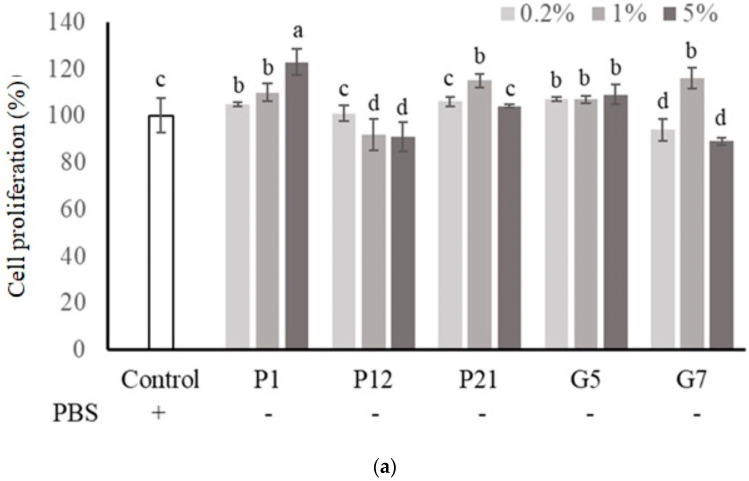

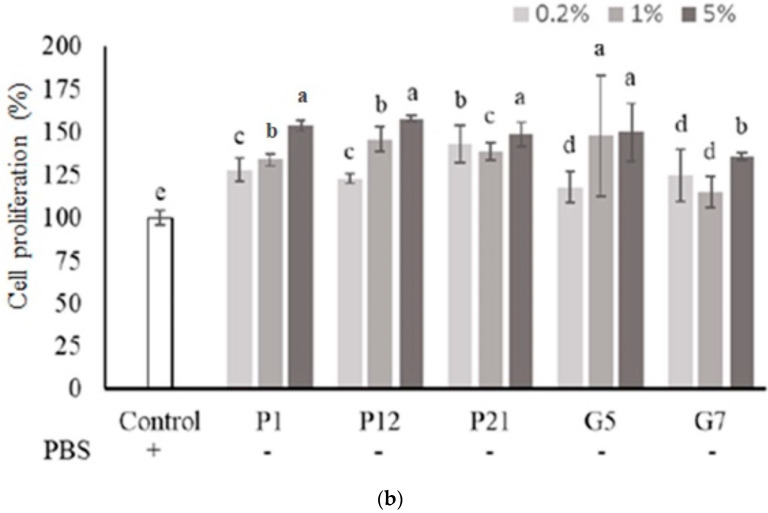
Effect of different cell-free supernatants (CFSs) on cell proliferation of Caco-2 cells. (**a**) Cell culture with 10% fetal bovine serum or (**b**) serum-free medium. Results are expressed as mean ± S.E.M. (n = 3). The different alphabetical superscripts indicate statistical significance (*p* < 0.05). Values represent mean ± SD (n = 3).

The selected CFSs from preliminary screening were evaluated their effect on intestinal epithelial cell proliferation at three concentrations (0.2%, 1%, and 5%) with or without serum. The results showed that P1, P21, and G5 had a better effect on promoting the proliferation of Caco-2 cells in the presence serum (Figure 1a). Additionally, in a serum-free medium (Figure 1b), Caco-2 cell proliferation was significantly increased after the addition of CFS in all treatments.

### 3.3. Effect of Selected CFSs on the Viability of DSS-Damaged Intestinal Epithelial Cells

**Figure 2:**
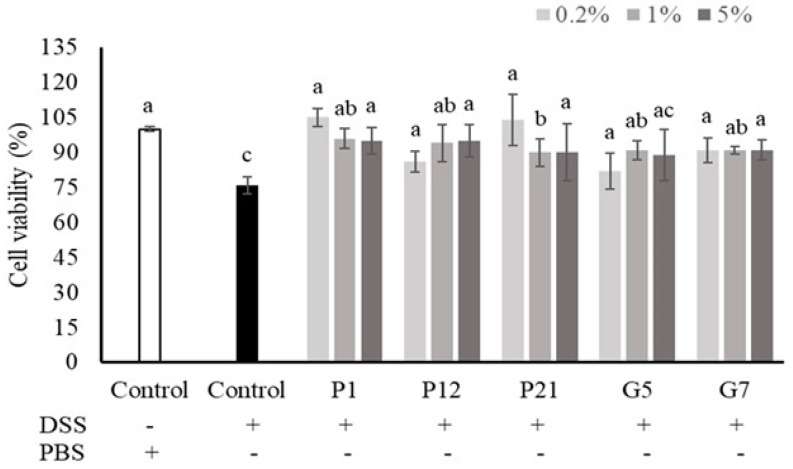
Effects of different cell-free supernatants (CFSs) on dextran sodium sulfate (DSS)-induced Caco-2 cell death. Results are expressed as mean ± S.E.M. (n = 3). The different alphabetical superscripts indicate statistical significance (*p* < 0.05). Values represent mean ± SD (n = 3).

The intestinal epithelial cells could be damaged upon DSS treatment, while a decrease in viability was observed (Figure 2). Our results demonstrated that co-culturing with CFSs from all selected strains could restore the cell death caused by DSS treatment, which showed the protective effects of the strains against chemical damage.

### 3.4. Effect of Selected CFSs on Transepithelial Electrical Resistance (TEER) of Caco-2 Cell Monolayers

**Figure 3:**
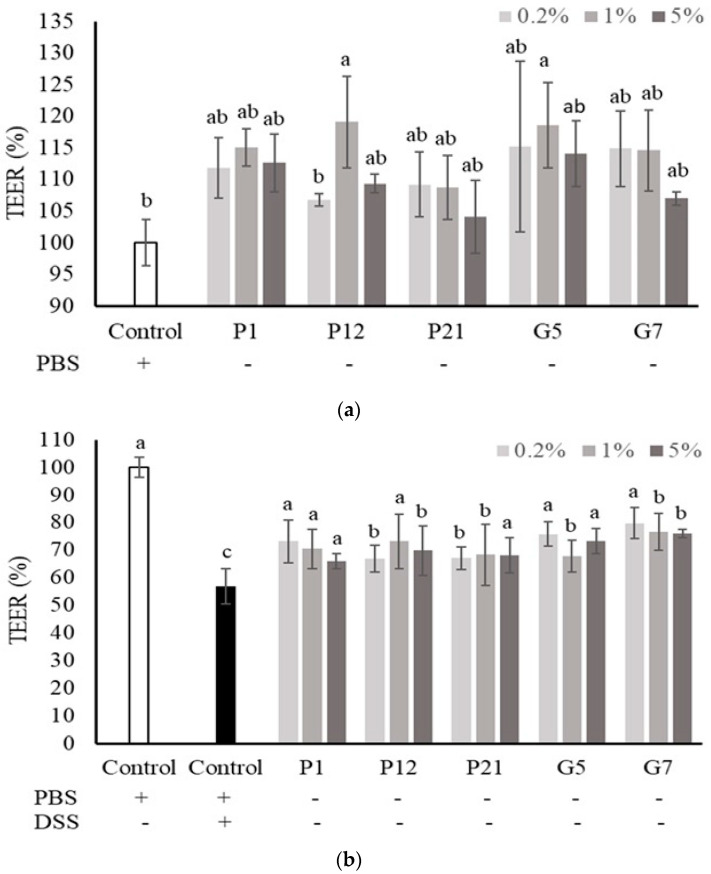
Effect of different cell-free supernatants (CFSs) on transepithelial electrical resistance (TEER) value of a Caco-2 monolayer. (**a**) TEER values were measured after Caco-2 monolayer treated with indicated CFSs for 24 h. (**b**) TEER values were measured after Caco-2 monolayer treated with indicated CFSs and 3% DSS for 72 h. Results are expressed as mean ± S.E.M. (n = 3). The different alphabetical superscripts indicate statistical significance (*p* < 0.05). Values represent mean ± SD (n = 3).

The Caco-2 cells were cultured onto a permeable membrane insert support to develop an intestinal cell monolayer with well-differentiated intestinal cells and matured barrier function. In this cell culture system, the TEER value was used to speculate the intestinal epithelial barrier function in vitro. The results showed that all the selected CFSs could increase the TEER value, but not significantly (Figure 3a), except for P12 and G5 at 1%, which could enhance TEER significantly when compared to the non-treated control group. However, the decrease in TEER value upon DSS treatment was significantly attenuated when co-culturing with all the selected CFSs (Figure 3b), indicating that the CFSs had a protective effect on the intestinal epithelial cell barrier under damaged conditions.

### 3.5. Effects of Selected CFSs on DSS-Induced Colitis in Mice

The candidate bacteria strains, P1, P12 and G7, were further selected to be evaluated in DSS-induced colitis mouse model, depending on the in vitro evaluation performed in intestinal epithelial Caco-2 cells described above (Figure 1, Figure 2 and Figure 3). In addition, because P1 and G5 (*L. salivarius*), as well as P12 and P21 (*L. gasseri*), were the same species, we finally selected P1 and P12 as representatives of their respective species.

**Figure 4:**
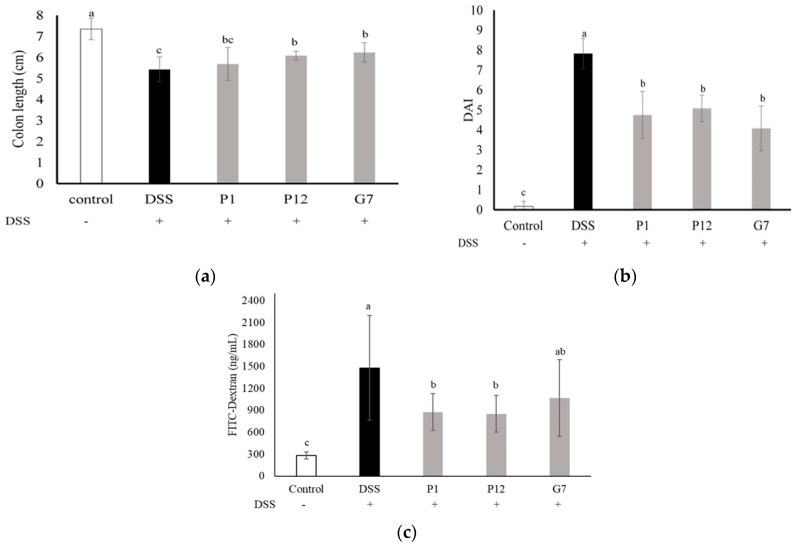
Effect of different cell-free fermentation supernatants (CFSs) on (**a**) disease activity index (DAI), (**b**) colon length, and (**c**) serum fluorescein isothiocyanate-dextran (FITC-D) level, in a dextran sodium sulfate (DSS)-induced colitis mouse model. Results are expressed as mean ± S.E.M. (n = 10). The different alphabetical superscripts indicate statistical significance (*p* < 0.05).

In the DSS-induced colitis mouse model, the body weight, fecal consistency, and occult blood level of the mice were measured and recorded daily and presented as the disease activity index (DAI) to monitor the degree of colitis. The highest DAI, indicating the most severe clinical symptoms in colitis, was seen in the DSS group, while the mice treated with P1, P12, and G7 CFSs showed significantly (*p* < 0.05) lower DAI values (Figure 4b). The length of the colon obtained after the mice were sacrificed was also measured. The DSS-induced colon shortening was significantly (*p* < 0.05) restored in mice treated with P12 and G7 (Figure 4a). The in vivo intestinal barrier function was monitored by using FITC-D, a widely employed permeability tracking reagent, to assess intestinal permeability in mice. The concentration of FITC-D in the sera of mice treated with DSS was significantly higher than that in other groups (Figure 4c), indicating that DSS caused intestinal damage and disrupted barrier function, resulting in increased intestinal permeability. In contrast, the concentration of FITC-D in the groups treated with CFSs was lower, with P1 and P12 showing significantly (*p* < 0.05) lower concentrations of FITC-D in the serum.

**Figure 5:**
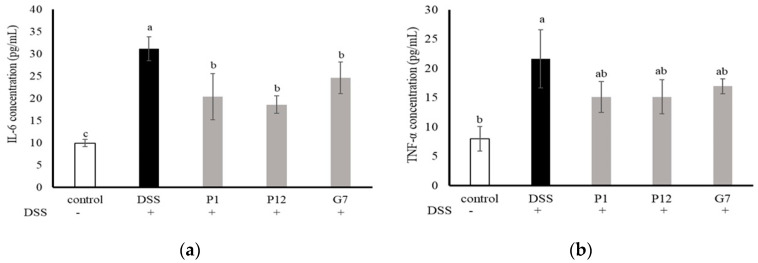
Effect of different cell-free supernatants (CFSs) on serum levels of (**a**) interleukin-6 (IL-6) and (**b**) tumor necrosis factor-α (TNF-α) in a dextran sodium sulfate (DSS)-induced mouse colitis model. Results are expressed as mean ± S.E.M. (n = 10). The different alphabetical superscripts indicate statistical significance (*p* < 0.05).

The levels of interleukin-6 (IL-6) and tumor necrosis factor-α (TNF-α) in the serum were measured to investigate the ability of the selected CFSs to reduce the level of pro-inflammatory cytokines in mice with DSS-induced colitis (Figure 5). The results showed that the concentrations of both IL-6 and TNF-α in the serum were significantly higher in the DSS group, indicating a more severe degree of inflammation. In the three groups fed with indicated CFS, the serum levels of IL-6 were significantly (*p* < 0.05) lower (Figure 5a), while the concentrations of TNF-α were lower but did not show statistically significant differences (Figure 5b). Overall, these findings suggest that the cell-free fermentation supernatant may reduce inflammation in the colons of mice with DSS-induced colitis.

**Figure 6:**
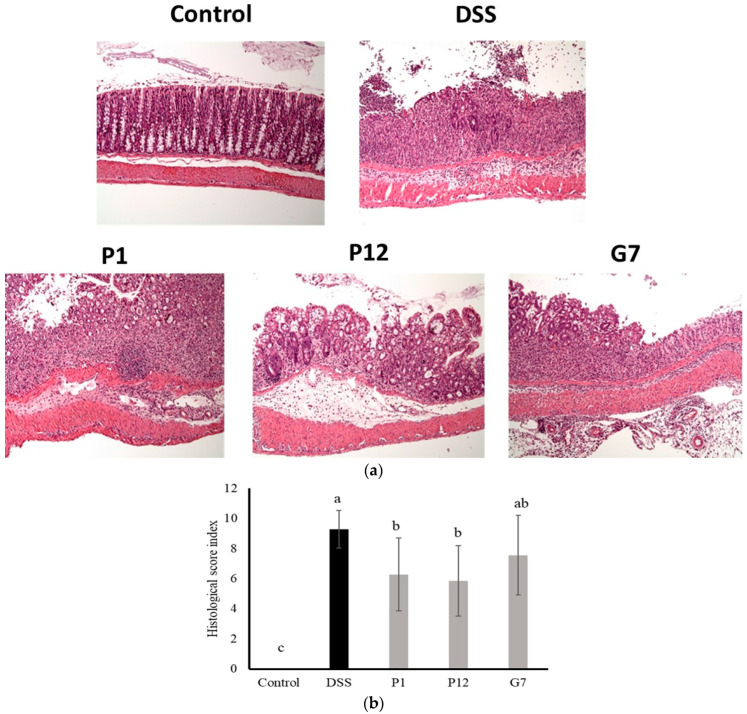
Representative histological photographs (**a**) and quantitative histological score (**b**) of a distal colon section from dextran sodium sulfate (DSS)-induced colitis mice administered with cell-free fermentation supernatants (CFSs). The images were captured at a magnification fold of 100×. Results are expressed as mean ± S.E.M. (n = 10). The different alphabetical superscripts indicate statistically significance (*p* < 0.05).

The results of the histological evaluation showed that normal mice had clear crypts and intact epithelial structure, indicating healthy colon tissue. In contrast, the colon tissue of DSS-induced colitis mice displayed a significant infiltration of inflammatory cells, resulting in an increase in abnormal epithelial cells and the development of irregularly shaped crypts, a common feature of colitis (Figure 6a). Severe infiltration led to the erosion of the lamina propria, leading to a loss of intact epithelial structure. In contrast, colon tissue from mice fed with CFSs from P1, P12, or G7 exhibited a lower density of inflammatory cell infiltration and a more preserved epithelial structure compared to the DSS group. The histopathological comprehensive scoring results (Figure 6b) showed that both P1 and P12 treatments had significantly (*p* < 0.05) lower scores compared to that in the DSS group, indicating a lower degree of inflammatory damage to intestinal tissue. In addition, the G7 group also showed a lower histopathological score, but this was not statistically significant.

### 3.6. Effects of Selected CFSs on Weaned Piglets

The selected strains, including P1, P12, and G7, were proven to show protective effects on the DSS-induced mouse colitis model in the current study. We further used a weaned piglet model to evaluate the possible application to pig growth upon weaning stress. When compared to the antibiotic supplementation group, after 6 weeks of feeding without antibiotic supplementation when weaning, the piglets showed significantly higher feed-to-gain ratios (F:G) and a lower feed conversion rate (FCR), indicating an inefficient growth performance in piglets (Table 1). However, the piglets in the low-dose G7 CFS powder group had significantly higher average daily gain (ADG) compared to the control group, and no significant difference was observed compared to the antibiotic group. In contrast, the piglets in the group supplemented with P12 CFS powder had a lower average daily gain and significantly lower average daily feed intake (ADFI), suggesting that it may have reduced the palatability of the feed for the piglets. There was no significant difference in F:G between the CFS feeding group and the antibiotic group, and the G7 low-dose CFS group had the best performance.

Additionally, the levels of pro-inflammatory cytokines, inflammatory indicator MPO activity, and endotoxins in serum were further analyzed in the weaned piglet model. In the results of cytokines in piglet serum collected after the end of the experiment, the addition of P1, P12, and low-dose G7 CFS powder to the feed increased the anti-inflammatory cytokine IL-10 in serum compared to the control group and the antibiotic group, indicating improved resistance to intestinal barrier damage caused by weaning (Figure S5a). There was no significant difference in the serum IL-18 level among all groups, but the level of the antibiotic group was higher (Figure S5b). Similarly, there were no significant differences among all groups in serum MPO activity (Figure S5c). Of the utmost significance, the serum endotoxin levels were markedly lower (*p* < 0.05) in all groups treated with CFSs compared to the untreated control group (Table 2). This indicates an enhancement in intestinal barrier function resulting from the administration of CFSs, which effectively guard against the entry of intestinal endotoxins during weaning stress, preventing leakage from the gut.

Overall, from the results of growth performance and serum endotoxin level, the supplementation of selected CFS powder in feed, including that from P1, P12, and G7 strains, could improve the growth efficiency and intestinal barrier function compared to the control group, and the results were not significantly different from those of the antibiotic group, which showed the possible potential of the CFSs to replace antibiotic use in the pork industry.

## 4. Discussion

This study focused on screening and applying bacterial CFS as a postbiotic. Intestinal epithelial cell models assessing viability, proliferation, and monolayer barrier function were employed for the initial screening of potential probiotic strains with positive effects. The in vivo effects of the CFSs from selected strains were further evaluated by using a DSS-induced colitis mouse model as well as a weaned piglet model. Results indicated that *L. salivarius* P1, *L. gasseri* P12, and *L. reuteri* G7, selected from 34 candidate strains, could ameliorate mouse colitis and counteract the decrease in growth performance induced by weaning in piglets. This study provides a systematic screening and evaluation platform for isolating potential probiotic strains and their postbiotics. To our knowledge, this study is the first to select potential probiotic strains for weaned piglets using an intestinal epithelial cell model combined with a DSS-induced colitis mouse model. This approach may streamline development and research, offering a more precise methodology for probiotic evaluation.

The cell line Caco-2 is a commonly used intestinal epithelial cell model due to its ability to exhibit various characteristics of intestinal epithelial cells when grown in cell culture [29]. The survival rate of Caco-2 cells upon different CFS treatments was analyzed in both a 10% serum medium and a serum-free medium for taking the first step to screen potential probiotic strains (Figures S1 and S2). The exposure of cells to a serum-free medium may lead to a loss of cell activity, inhibition of differentiation, and acceleration of apoptosis [30,31]. Therefore, the serum-free medium is suitable for use as a rapid method to screen whether biological samples could affect cell growth or death. For example, adding growth factors, protein hydrolysates, amino acids, and other supplements to a serum-free medium can aid in cell recovery to a level similar to that seen in a serum-supplemented medium [32]. This suggests that the CFSs from selected strains may contain necessary nutrients or growth factors for cell growth, and five CFSs, which were from *L. salivarius* P1, *L. gasseri* P12, *L. gasseri* P21, *L. salivarius* G5, and *L. reuteri* G7, respectively, were chosen for further evaluation due to their positive effects on the increase in cell proliferation in serum-free culture conditions (Figure 1b). Additionally, these selected CFSs could also show protective effects on Caco-2 cells against DSS-induced cell death (Figure 2). Based on these findings, the addition of selected CFSs could ameliorate the harsh conditions in the intestinal microenvironment, including a lack of nutrients and the presence of toxic substances, to protect intestinal epithelial cells.

Caco-2 cells can be cultured on a permeable membrane to differentiate into a columnar absorptive cell type, developing into a polarized monolayer that expresses a brush border on the apical surface with typical intestinal enzymes and transporters. This monolayer forms dense intercellular junctional complexes constituting a tight epithelium contributing to barrier function [29]. In this intestinal epithelial cell monolayer culture system, TEER is a commonly used parameter to monitor intestinal barrier function. By measuring the TEER value, the electrical impedance of the cells can be determined based on the continuous current passing through the trans-cellular and para-cellular pathways, allowing for the evaluation of the barrier function of the cells [33,34]. In our study, the selected CFSs showed a limited effect on the increase in TEER value in the Caco-2 monolayer (Figure 3a). However, all selected CFSs demonstrated significant effects on the Caco-2 monolayer against a DSS-induced decrease in TEER value (Figure 3b), along with the restoration of cell proliferation under the same challenge (Figure 2). This indicates that the CFSs have the potential to be applied to intestinal disorders.

Except for use in cell culture, DSS is the most widely used chemical agent for inducing colitis in mice [23,35]. Here, a preventive model was designed to investigate the protective effect of the selected CFSs on the DSS-induced intestinal damage *in vivo*. Based on the consideration of species variety and the results of Caco-2 cell culture experiments (Figure 1, Figure 2 and Figure 3), the CFS from three indicated strains, including *L. salivarius* P1, *L. gasseri* P12, and *L. reuteri* G7, were further selected for evaluating in the DSS colitis model in mice. The results obtained in our study showed that all selected CFSs of the three strains could ameliorate DSS-induced colitis in mice, including the improvement of the DAI (Figure 4a), restoration of colon length shortening (Figure 4b), improvements in intestinal barrier permeability (Figure 4c), and histopathological evaluation of damaged colon (Figure 6). In addition, systemic inflammation was also improved, as serum pro-inflammatory cytokines were downregulated in the presence of the CFS (Figure 5). The intestinal mucosal barrier plays a crucial role in preventing the entry of microorganisms and bacterial toxins into the systemic circulation. However, when intestinal inflammation occurs, the damaged barrier may allow increased permeability, which is a pathological feature of colitis [36]. Compared to those using the live probiotic bacteria to ameliorate colitis, there are few studies aimed at using postbiotics. Carlsson et al. [37] found that *Faecalibacterium prausnitzii* CFS could protect mice against DSS-induced colitis by producing butyrate, which could provide energy for intestinal epithelial cells and influence gene expression involving in cell proliferation. Similarly, heat-inactivated *Lactobacillus brevis* SBC8803 reduced inflammation and improved the intestinal barrier by activating the p38 MAPK pathway and downregulating the rapid expression of pro-inflammatory cells such as TNF-α and IL-1β [38]. These findings suggest that even in the absence of viable bacteria, CFS or other postbiotics can still protect intestinal epithelial cells through bioactive components produced by the metabolism of probiotics, improve the intestinal barrier function, and further maintain intestinal homeostasis.

Antibiotic use in livestock farming as an antibiotic growth promotor (AGP) has contributed to the spread of antibiotic-resistant microbes in animals and humans, which is a major public health concern. Weaning can cause stress and changes in nutrition for piglets, which can affect their growth [39]. There is a growing interest among researchers in seeking alternatives to antibiotics to enhance piglet growth during weaning without compromising their intestinal barriers [40]. Our study showed that adding CFS powder of *L. reuteri* G7 to the feed for 6 weeks in a weaned piglet model improved the F:G ratio and FCR compared to the control group without antibiotic supplement. The performance was not significantly different from that of the antibiotic group (Table 1), indicating that the selected *L. reuteri* G7 CFS has the potential to replace the use of antibiotics during weaning. The most possible mechanism is that the CFS could improve the intestinal barrier function, as a decrease in serum endotoxin levels was observed (Table 2), which was consistent with the results from the DSS colitis mouse model (Figure 4c). These findings are consistent with previous research demonstrating that adding *L. plantarum* metabolites to the diet of weanling piglets can replace the use of antibiotics to improve weight loss caused by weaning and result in growth performance [41].

IL-10 acts as an anti-inflammatory cytokine that can attenuate tight junction permeability defects and have effects on epithelial repair after injury, including increased expression of extracellular matrix proteins and cell surface integrins, enhanced epithelial cell migration, and decreased para-epithelial cell permeability [42]. In the present study, incorporating P1, P12, and low-dose G7 concentrated fermentation supernatant powder into the feed exhibited a tendency to elevate the porcine serum IL-10 level compared to both the control group and the antibiotic group (Figure S5a). While the increase was not statistically significant, it suggests the potential to ameliorate intestinal barrier damage induced by weaning. On the other hand, IL-18, also known as an IFN-γ activator, is a cytokine that plays a crucial role in regulating innate and acquired immune responses [43]. However, the production of IL-18 by hyperactivated macrophages can induce a pro-inflammatory response by enhancing IFN-γ gene expression in T cells and synergizing with IL-12 [44]. In our study, there was no significant difference in the expression of IL-18 among the experiment groups, which indicated that no severe systemic damage occurred in piglets (Figure S5b).

Intestinal inflammation leads to intestinal oxidative stress and can cause damage to the mucosal barrier [45]. MPO is a marker of neutrophil activity and has been shown to be positively correlated with the number of neutrophils in mammals [46]. Studies have also demonstrated that MPO activity is increased in response to heat stress and is associated with intestinal permeability in piglets [47] and with colitis in a piglet model [48]. Hence, the activity of myeloperoxidase (MPO) serves as an indicator of the integrity of the intestinal mucosal barrier [48]. In this study, it was observed that the antibiotic group exhibited a slightly elevated, albeit statistically nonsignificant, serum level of MPO activity when compared to both the control group and the group treated with various fermentation supernatant powders (Figure S5c). This may be due to the impact of antibiotics on the balance of intestinal microbiota, resulting in increased barrier permeability [49]. The increase in intestinal barrier permeability was also shown in the current study, where the piglets from the control group without any treatment had the highest serum endotoxin level in the 6-week experimental period (Table 2). During the early stages of intestinal epithelial barrier damage, bacteria and their by-products can translocate across the basement membrane and trigger inflammation. Endotoxin, also known as lipopolysaccharide (LPS), is a major component of the cell wall of Gram-negative bacteria and is a potent pro-inflammatory factor that can cross the intestinal barrier into systemic circulation [50]. In the current study, antibiotic- and CFS-treated piglets had lower serum levels of endotoxin when compared to the non-treated control piglets (Table 2). This is likely due to the fact that antibiotics indiscriminately destroy the internal symbiotic bacteria, leading to relatively low numbers of bacteria in the body [49] and, consequently, lower serum endotoxin concentrations. The four CFS groups had significantly lower serum endotoxin levels compared to the control group and did not differ significantly from the antibiotic group. These results suggest that the addition of CFS powders to feed may improve intestinal barrier function in piglets and have a similar effect to antibiotics in promoting health. Our results were similar to Wang et al. [51], who found that feeding weaned piglets a high-moisture corn silage diet supplemented with *L. acidophilus* and *Pediococcus acidilactici* resulted in a decrease in serum endotoxin levels and improved intestinal barrier function compared to the control group. Although the added probiotic strains were not detected in the feces, indicating that they did not colonize the intestine, they still appeared to regulate the intestinal microbiota and improve growth performance through the production of short-chain fatty acids and lactic acid.

Generally, the results of this study on the supplementation of CFS as a postbiotic in weaned piglets suggest that it has a positive effect on the health of weaned piglets, including growth performance, serum cytokine concentration, and endotoxin concentration. The bacterial metabolites in the CFS may regulate these effects through various mechanisms and reduce the negative impact of weaning on piglet health.

## 5. Conclusions

In this study, we selected three CFSs from different probiotic strains, namely *L. salivarius* P1, *L. gasseri* P12, and *L. reuteri* G7, using Caco-2 intestinal epithelial cells. All CFSs demonstrated the promotion of cell proliferation and an improvement in barrier function. Further in vivo evaluation was conducted using a DSS-induced colitis mouse model, where CFSs from P1, P12, and G7 all exhibited improvements in disease symptoms, shortened colon length, disruption of intestinal permeability, histological damages, and reduced serum pro-inflammatory cytokine levels. In weaned piglets, the addition of CFS powder to the feed resulted in similar growth performance compared to antibiotic-treated piglets, with lower serum endotoxin levels. Notably, CFS from G7 showed the best performance. Overall, we employed a sequential selection platform involving cell, mouse, and pig models to identify and evaluate potential CFSs from probiotic strains as postbiotics. The CFS of *L. reuteri* G7 was ultimately chosen as a postbiotic in feed additives to enhance growth performance and barrier function in weaned piglets. However, further research focused on intestinal protection is recommended to broaden the application of the selected CFSs from this study.

## Figures and Tables

**Table 1:** Effects of fermentation supernatant powder on growth performance of weaned piglets.

		Control	Antibiotic	P1	P12	G7 (L)	G7 (H)
Feed-to-gain ratio (F:G)	W1	0.78 ± 0.12	0.83 ± 0.08	0.64 ± 0.03	0.67 ± 0.05	0.70 ± 0.02	0.75 ± 0.05
	W2	0.65 ± 0.03	0.72 ± 0.01	0.65 ± 0.04	0.53 ± 0.16	0.64 ± 0.03	0.66 ± 0.05
	W3	0.64 ± 0.01 ^a^	0.52 ± 0.01 ^b^	0.52 ± 0.04 ^b^	0.54 ± 0.02 ^b^	0.58 ± 0.03 ^ab^	0.59 ± 0.03 ^ab^
	W4	0.60 ± 0.03	0.61 ± 0.02	0.60 ± 0.02	0.60 ± 0.01	0.60 ± 0.02	0.64 ± 0.02
	W5	0.57 ± 0.02	0.53 ± 0.01	0.52 ± 0.01	0.55 ± 0.01	0.57 ± 0.03	0.53 ± 0.01
	W6	0.49 ± 0.04 ^b^	0.63 ± 0.02 ^a^	0.55 ± 0.03 ^ab^	0.58 ± 0.05 ^ab^	0.62 ± 0.03 ^a^	0.57 ± 0.02 ^ab^
Feed conversion rate (FCR)	W1	1.36 ± 0.16 ^ab^	1.19 ± 0.13 ^b^	1.57 ± 0.06 ^a^	1.53 ± 0.12 ^ab^	1.42 ± 0.04 ^ab^	1.31 ± 0.10 ^ab^
	W2	1.55 ± 0.06	1.40 ± 0.02	1.56 ± 0.09	1.53 ± 0.09	1.57 ± 0.08	1.55 ± 0.12
	W3	1.56 ± 0.04 ^b^	1.93 ± 0.03 ^a^	1.96 ± 0.14 ^a^	1.86 ± 0.07 ^a^	1.74 ± 0.09 ^ab^	1.72 ± 0.10 ^ab^
	W4	1.67 ± 0.08	1.65 ± 0.07	1.68 ± 0.05	1.67 ± 0.04	1.68 ± 0.07	1.57 ± 0.06
	W5	1.76 ± 0.06	1.91 ± 0.05	1.93 ± 0.05	1.81 ± 0.04	1.78 ± 0.08	1.88 ± 0.02
	W6	2.10 ± 0.17 ^a^	1.57 ± 0.03 ^b^	1.84 ± 0.11 ^ab^	1.76 ± 0.15 ^ab^	1.62 ± 0.08 ^b^	1.76 ± 0.07 ^ab^

Results are expressed as mean ± S.E.M. (n = 8). The different alphabetical superscripts indicate statistical significance (*p* < 0.05). P1: 5% CFS powder of *Ligilactobacillus salivarius* P1; P12: 5% CFS powder of *Lactobacillus gasseri* P12; G7 (L): 0.5% CFS powder of *Limosilactobacillus reuteri* G7; G7 (H): 5% CFS powder of *Limosilactobacillus reuteri* G7.

**Table 2:** Effect of cell-free fermentation supernatant powder on serum endotoxin levels in weaned piglets.

		Control	Antibiotic	P1	P12	G7 (L)	G7 (H)
Endotoxin (EU/mL)	Week 0	2.710	2.553	2.502	3.893	2.474	2.539
	Week 6	6.070 ^a^	1.715 ^b^	3.170 ^ab^	2.243 ^b^	1.974 ^b^	1.819 ^b^

Results are expressed as mean ± S.E.M. (n = 8). The different alphabetical superscripts indicate statistical significance in the same row (*p* < 0.05). P1: 5% CFS powder of *Ligilactobacillus salivarius* P1; P12: 5% CFS powder of *Lactobacillus gasseri* P12; G7 (L): 0.5% CFS powder of *Limosilactobacillus reuteri* G7; G7 (H): 5% CFS powder of *Limosilactobacillus reuteri* G7.

## Data Availability

Data is contained within the article or Supplementary Materials.

## Supplementary Materials

The following supporting information can be downloaded at: https://www.mdpi.com/article/10.3390/nu16081138/s1, Figure S1: Preliminary screening of 34 potential cell-free supernatants (CFSs) on improving the cell viability of Caco-2 cells cultured in medium (a) with or (b) without 10% fetal bovine serum (FBS). Values represent mean ± SD (n = 3); Figure S2: Effects of 18 different cell-free supernatants (CFSs) on the cell viability of Caco-2 cells. (a–c) 0.2%, 1%, and 5% of CFS in cell culture with 10% FBS medium. (d–f) 0.2%, 1%, and 5% of CFS in cell culture with serum-free medium. Values represent mean ± SD (n = 3); Figure S3: Effect of cell-free fermentation supernatants (CFSs) on transepithelial electrical resistance (TEER) of the intestinal epithelial monolayer. (a–c) Cells were treated with PBS or 0.2%, 1%, and 5% CFS, respectively, at 0 h. TEER was measured and analyzed over 24 h. (d–f) Cells were pre-treated with 3% DSS and then treated with PBS or a concentration of 0.2%, 1%, and 5% CFS, respectively, at 0 h. TEER was measured and analyzed over 36 h. Values represent mean ± SD (n = 3). Values represent mean ± SD (n = 3); Figure S4: Effect of different cell-free fermentation supernatants (CFSs) on body weight changes in a dextran sodium sulfate (DSS)-induced colitis mouse model. Results are expressed as mean ± S.E.M. (n = 10). The different alphabetical superscripts indicate statistical significance (*p* < 0.05); Figure S5: Effect of cell-free fermentation supernatant powders on serum cytokine concentration and MPO activity in weaned piglets. (a) IL-10, (b) IL-18, and (c) MPO activity. Values represent mean ± SD (n = 8).
