# Supplementary material for: Selection of Fermentation Supernatant from Probiotic Strains Exhibiting Intestinal Epithelial Barrier Protective Ability and Evaluation of Their Effects on Colitis Mouse and Weaned Piglet Models
Source: Nutrients. 2024 Apr 12;16(8):1138. doi: 10.3390/nu16081138 (PMC11053620; doi:10.3390/nu16081138)
Supplement: Supplementary file 1 [file nutrients-16-01138-s001.zip › nutrients-2929860-supplementary.pdf]

Supplementary figures

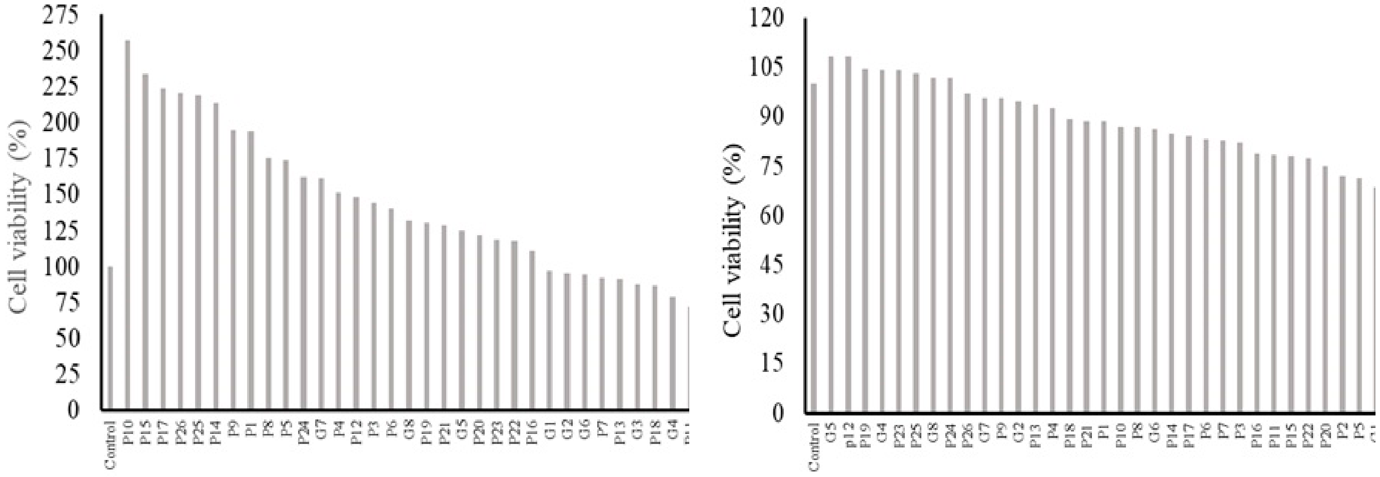

(a) (b)

Figure S1. Preliminary screening of 34 potential cell-free supernatant (CFS) on improving cell viability of Caco-2 cells cultured in medium (**a**) with or (**b**) without 10% fetal bovine serum (FBS). Values represented mean ± SD (n = 3).

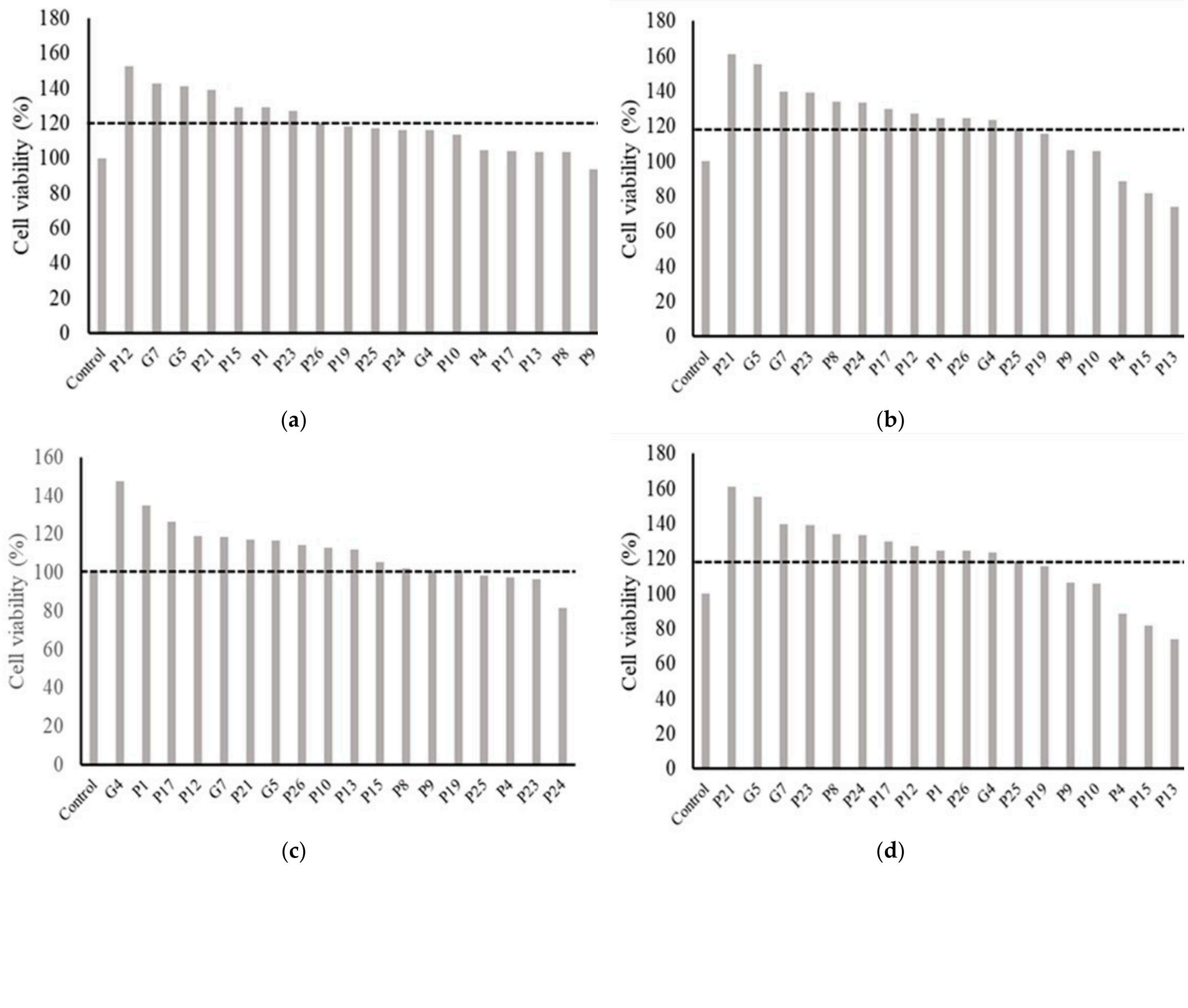

(a) (b)

(c) (d)

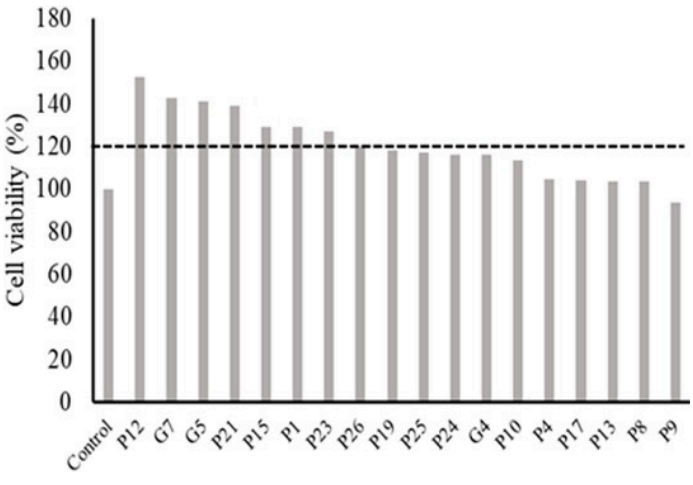

(e)

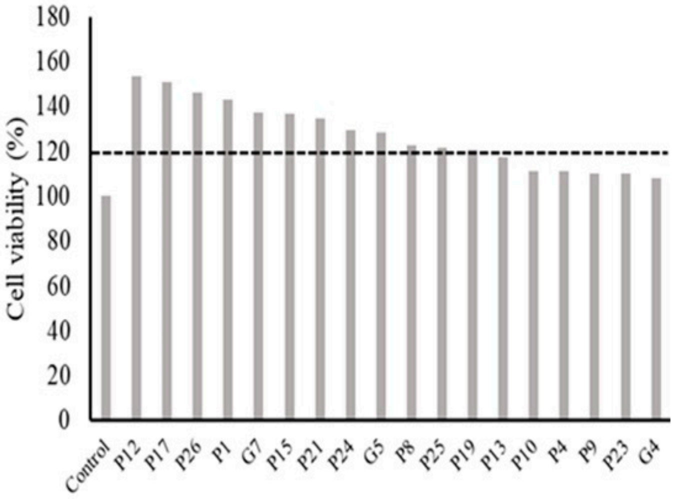

(f)

Figure S2. Effects of 18 different cell-free supernatants (CFS) on cell viability of Caco-2 cells. (**a–c**) 0.2%, 1% and 5% of CFS in cell culture with 10% FBS medium. (**d–f**) 0.2%, 1% and 5% of CFS in cell culture with serum-free medium. Values represented mean ± SD (n = 3).

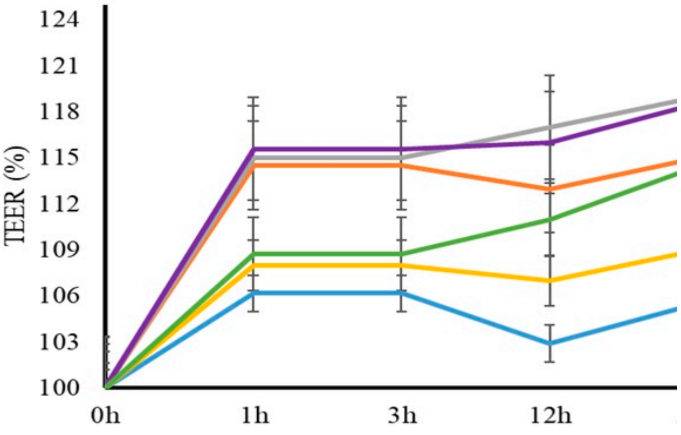

(a)

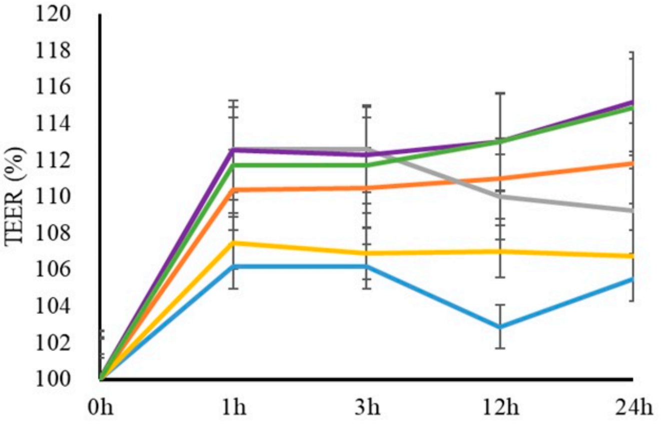

(b)

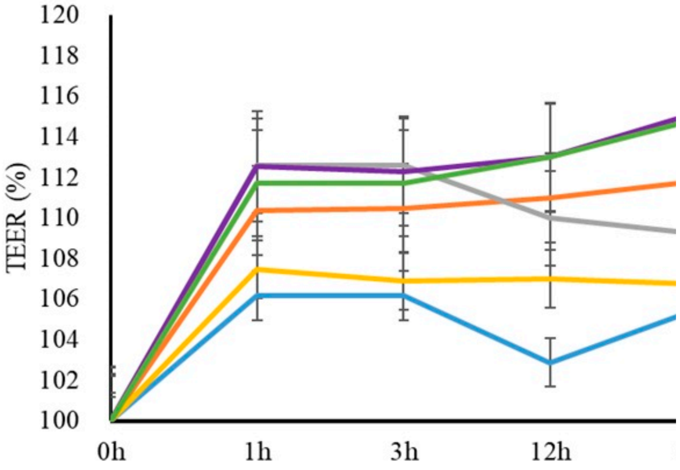

(c)

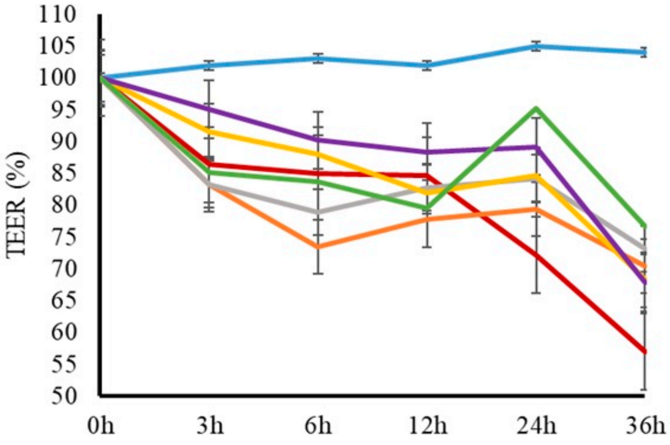

(d)

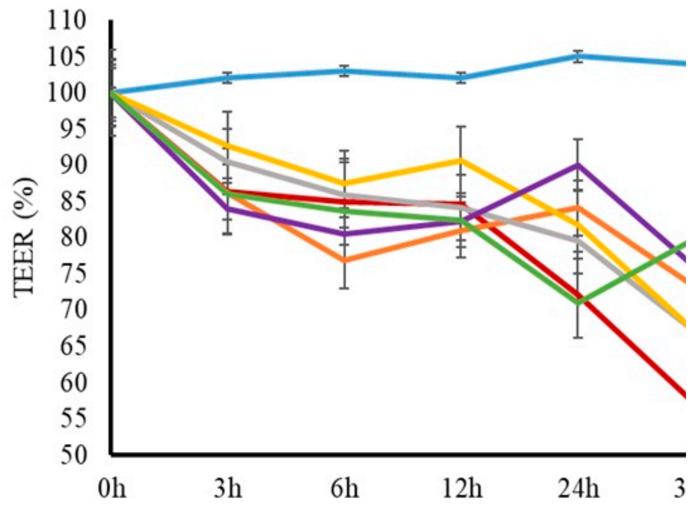

(**e**)

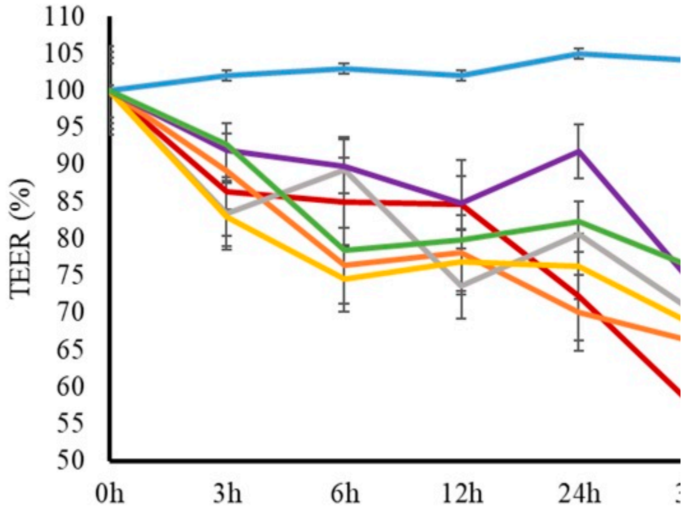

(**f**)

Figure S3. Effect of cell-free fermentation supernatants (CFS) on (transepithelial electrical resistance /TEER) of intestinal epithelial monolayer. (**a**–**c**) Cells were treated with PBS or 0.2%, 1%, and 5% CFS respectively at 0 h. TEER was measured and analyzed over 24h. (**d**–**f**) Cell were pre-treated with 3% DSS and then treated with PBS or concentration of 0.2%, 1%, and 5% CFS respectively at 0 h. TEER was measured and analyzed over 36 h. Values represented mean ± SD (n = 3). Values represented mean ± SD (n = 3).

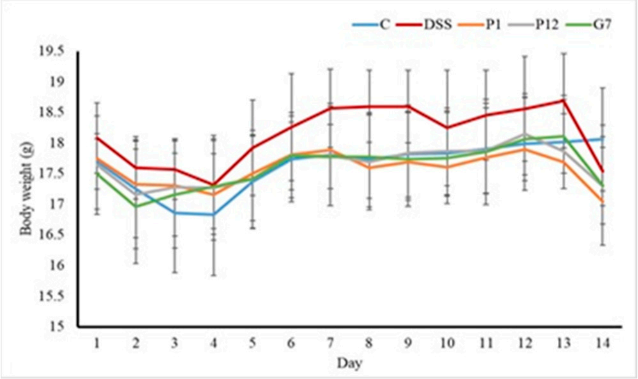

Figure S4. Effect of different cell-free fermentation supernatants (CFSs) on body weight changes in dextran sodium sulphate (DSS)-induced colitis mouse model. Results are expressed as mean ± S.E.M. (n = 10). The different alphabetical superscripts are found statistically significant ($P < 0.05$).

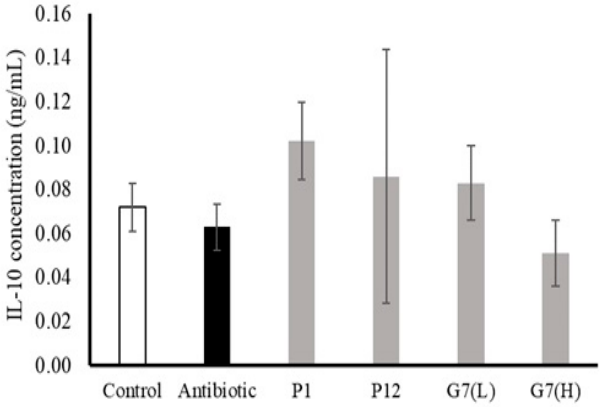

(**a**)

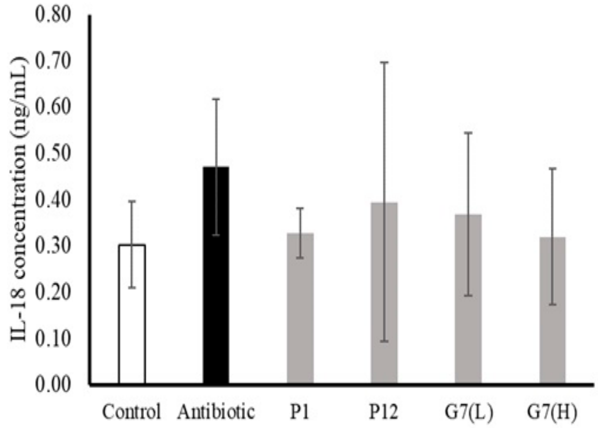

(**b**)

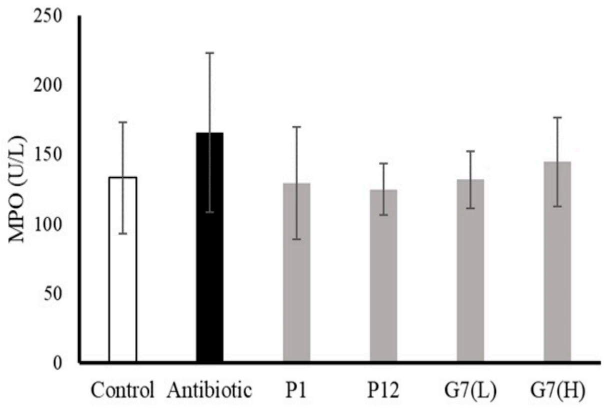

(**c**)

Figure S5. Effect of cell-free fermentation supernatants powder on serum cytokine concentration and MPO activity in weaned piglets. (**a**) IL-10, and (**b**) IL-18 (**c**) MPO activity. Values represented mean ± SD (n = 8).
